# Exploring the associations between gut microbiota composition and SARS-CoV-2 inactivated vaccine response in mice with type 2 diabetes mellitus

**DOI:** 10.1128/msphere.00380-24

**Published:** 2024-08-27

**Authors:** Long Liu, Xianzhen He, Jiaqi Wang, Moran Li, Xiuli Wei, Jing Yang, Gong Cheng, Weixing Du, Zhixin Liu, Xiao Xiao

**Affiliations:** 1 Department of Pathogen Biology, School of Basic Medical Sciences, Renmin Hospital, Hubei University of Medicine, Shiyan, China; 2 Institute of Virology, Shiyan Key Laboratory of Virology, Hubei University of Medicine, Shiyan, China; 3 Hubei Key Laboratory of Embryonic Stem Cell Research, Hubei University of Medicine, Shiyan, China; 4 Department of Children’s Medical Center, Renmin Hospital, Hubei University of Medicine, Shiyan, China; 5 New Cornerstone Science Laboratory, Tsinghua-Peking Joint Center for Life Sciences, School of Basic Medical Sciences, Tsinghua University, Beijing, China; Nanjing University of Chinese Medicine, Nanjing, Jiangsu, China

**Keywords:** SARS-CoV-2 inactivated vaccine, gut microbiota, lung microbiota, type 2 diabetes mellitus

## Abstract

Severe acute respiratory syndrome coronavirus 2 (SARS-CoV-2) vaccination is crucial for protecting vulnerable individuals, yet individuals with type 2 diabetes mellitus (T2DM) often exhibit impaired vaccine responses. Emerging evidence suggests that the composition of the host microbiota, crucial in immune regulation and development, influences vaccine efficacy. This study aimed to characterize the relationships between the SARS-CoV-2 inactivated vaccine and the host microbiota (specifically, gut and lung microbiota) of C57BL/6 mice with T2DM. Employing 16S rRNA metagenomic sequencing and ultra-high-performance liquid chromatography-mass spectrometry, we observed lower alpha diversity and distinct beta diversity in fecal microbiota before vaccination and in gut microbiota 28 days post-vaccination between T2DM mice and healthy mice. Compared with healthy mice, T2DM mice showed a higher Firmicutes/Bacteroidetes ratio 28 days post-vaccination. Significant alterations in gut microbiota composition were detected following vaccination, while lung microbiota remained unchanged. T2DM was associated with a diminished initial IgG antibody response against the spike protein, which subsequently normalized after 28 days. Notably, the initial IgG response positively correlated with fecal microbiota alpha diversity pre-vaccination. Furthermore, after 28 days, increased relative abundance of gut probiotics (*Bifidobacterium* and *Lactobacillus*) and higher levels of the gut bacterial tryptophan metabolite, indole acrylic acid, were positively associated with IgG levels. These findings suggest a potential link between vaccine efficacy and gut microbiota composition. Nonetheless, further research is warranted to elucidate the precise mechanisms underlying the impact of the gut microbiome on vaccine response. Overall, this study enhances our understanding of the intricate relationships among host microbiota, SARS-CoV-2 vaccination, and T2DM, with potential implications for improving vaccine efficacy.

**IMPORTANCE:**

Over 7 million deaths attributed to severe acute respiratory syndrome coronavirus 2 (SARS-CoV-2) by 6 May 2024 underscore the urgent need for effective vaccination strategies. However, individuals with type 2 diabetes mellitus (T2DM) have been identified as particularly vulnerable and display compromised immune responses to vaccines. Concurrently, increasing evidence suggests that the composition and diversity of gut microbiota, crucial regulators of immune function, may influence the efficacy of vaccines. Against this backdrop, our study explores the complex interplay among SARS-CoV-2 inactivated vaccination, T2DM, and host microbiota. We discover that T2DM compromises the initial immune response to the SARS-CoV-2 inactivated vaccine, and this response is positively correlated with specific features of the gut microbiota, such as alpha diversity. We also demonstrate that the vaccination itself induces alterations in the composition and structure of the gut microbiota. These findings illuminate potential links between the gut microbiota and vaccine efficacy in individuals with T2DM, offering valuable insights that could enhance vaccine responses in this high-risk population.

## INTRODUCTION

Since 2019, the emergence of severe acute respiratory syndrome coronavirus 2 (SARS-CoV-2) caused more than 775 million reported infection cases, leading to more than 7 million deaths until 6 May 2024 (1). The global development and deployment of SARS-CoV-2 vaccines have saved millions of lives by protecting vulnerable populations associated with increased risks of morbidity and mortality, including people with diabetes (2, 3). However, multiple studies have documented impaired antibody responses in patients with T2DM following SARS-CoV-2 vaccination (3–6).

Emerging evidence suggests that the variability in immune responses to vaccination may be linked to the gut microbiota (7). The human gastrointestinal tract hosts a diverse and dynamic community of microorganisms known as the gut microbiota (8). Through the production of various metabolites and modulation of the gut environment, the gut microbiota exerts influence across the human body, including on the immune response to vaccination (9, 10). For example, responders to a rice-based cholera toxin B subunit vaccination had a higher diversity in their gut microbiota compared to non-responders (11). In India and Malawi, infants receiving two doses of oral rotavirus vaccine showed a negative relationship between increased microbiota diversity and oral rotavirus vaccine immunogenicity (12). Specific gut microbes were significantly correlated with vaccine responses. For example, Jiang et al. found that gut *Sutterella* in infant rhesus macaques was positively correlated with human immunodeficiency virus vaccine-elicited antibody responses (13). Analysis of the gut microbiota in 122 Ghanaian infants revealed that gut *Streptococcus* abundance was positively correlated with seroconversion of rotavirus vaccines (14). Additionally, modification of the gut microbiota by antibiotics, probiotics, and fecal microbiota transplant (FMT) leads to varied vaccine immunogenicity (15–17). Different dietary fiber intakes can modulate SARS-CoV-2 vaccine response maturation (16). Administration of the oral probiotic *Lactobacillus plantarum* has been found to promote SARS-CoV-2 neutralizing antibodies (17). Furthermore, improved immune responses to SARS-CoV-2 vaccination have shown potential associations with the relative abundance of specific gut microbes, such as *Bifidobacterium adolescentis*, *Roseburia faecis*, *Clostridium leptum*, *Lactobacillus ruminis*, and *Ruminococcus torques* (16, 18, 19). Notably, a cohort study on inflammatory bowel disease patients receiving SARS-CoV-2 vaccines found that individuals with below-average antibody concentrations exhibited lower beta diversity of the gut microbiota (10). Hu et al. also discovered significant differences in gut microbiota composition between high- and low-response groups to SARS-CoV-2 vaccination (20). Thus, the gut microbiota may represent a significant factor influencing the immunogenicity of SARS-CoV-2 vaccines.

Available evidence suggests that T2DM is associated with the dysbiosis of the gut microbiota (21). Compared with healthy individuals, patients with T2DM exhibit lower overall alpha diversity of the gut microbiota and increased gut permeability, which may impact the host immune system (22). Additionally, certain gut metabolites, such as short-chain fatty acids (SCFAs), trimethylamine N-oxide, succinate, bile acids, and tryptophan-derived metabolites, showed close relationships to the development of T2DM (21). Furthermore, FMT can increase gut microbial diversity, improve clinical indicators, and inhibit chronic inflammation in pancreatic tissue in patients with T2DM (23, 24). Consequently, the gut microbiota has been proposed as a prospective therapeutic target for T2DM via the modulation of inflammation (22).

Previously thought to be sterile, recent research has revealed a low bacterial load in the lower respiratory tract of healthy individuals, now recognized as the lung microbiota (25). Dysbiosis of the lung microbiota has been linked to the progression of various diseases, including pneumonia, asthma, chronic obstructive pulmonary disease, lung cancer, and lung fibrosis (25). Moreover, the lung microbiota is associated with systemic and bronchial markers of inflammation, as well as basal immune responses in the lower lungs (26, 27). Recent studies have suggested that the immune responses to vaccines, such as influenza and SARS-CoV-2 vaccines, may be influenced by the lung microbiota (7). Given the intricate interplay between T2DM, the immune system, and the host microbiota (both gut and lung), we conducted an animal experiment to investigate the hypothesis that SARS-CoV-2 vaccination could alter host microbiota composition and metabolites, and the dysbiosis of host microbiota triggered by T2DM may attenuate the immune response to vaccination.

## RESULTS

### T2DM influences the initial immune response to vaccination

**Fig 1:**
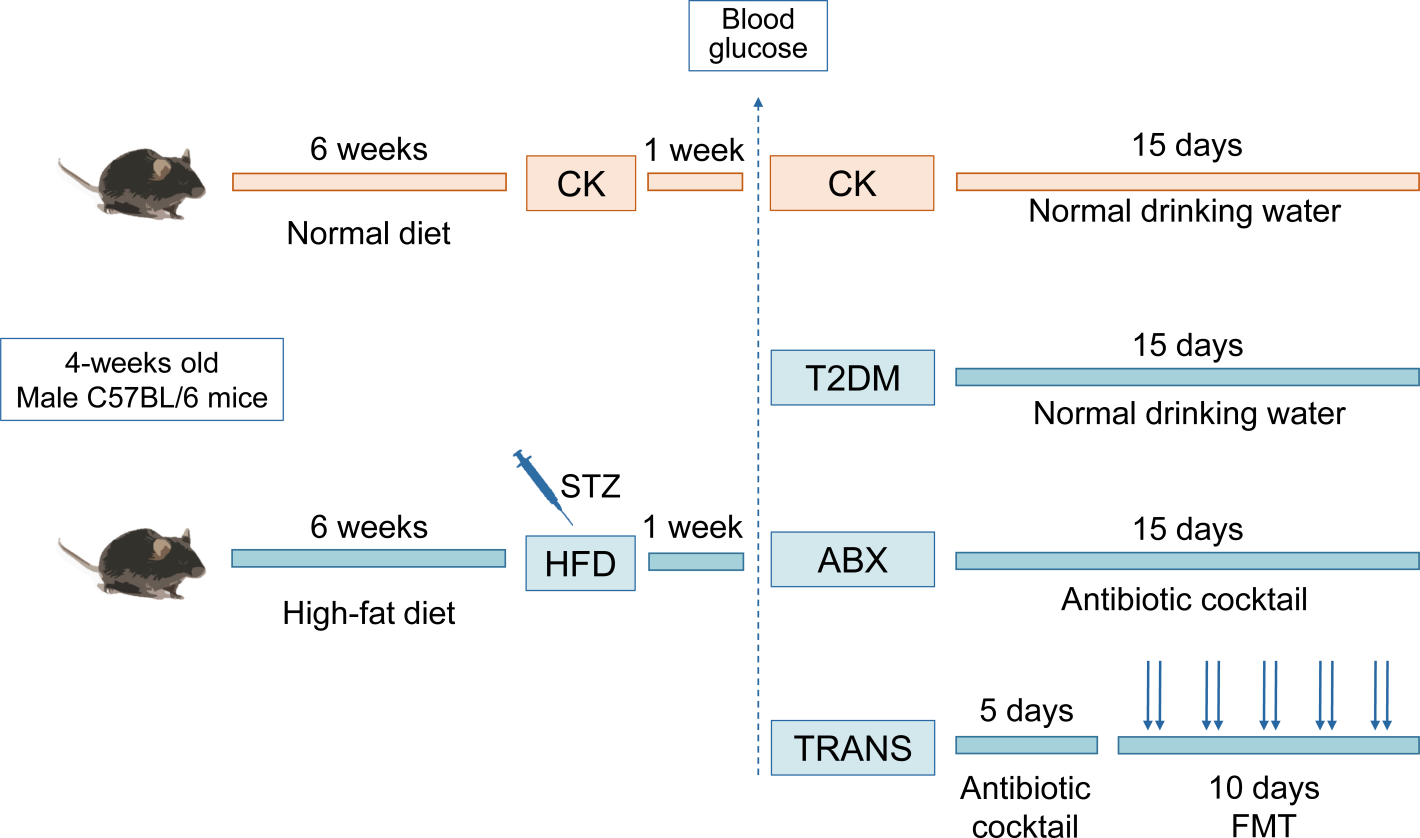
Workflow of animal experiments. The T2DM mouse model was established by feeding a high-fat diet for 6 weeks and injecting STZ. CK mice (*n* = 10) were injected with the same amount of citrate–phosphate buffer as the normal control. After confirming blood glucose levels, T2DM mice were divided into three groups: T2DM group (*n* = 15, provided with normal drinking water *ad libitum*), ABX group (*n* = 14, administered water with antibiotic cocktail regime *ad libitum* for 15 days), and TRANS group (*n* = 16). The TRANS group received drinking water with antibiotic cocktail *ad libitum* for 5 days, followed by oral gavage of donor microbiota from the CK group every other day for 10 days.

**Fig 2:**
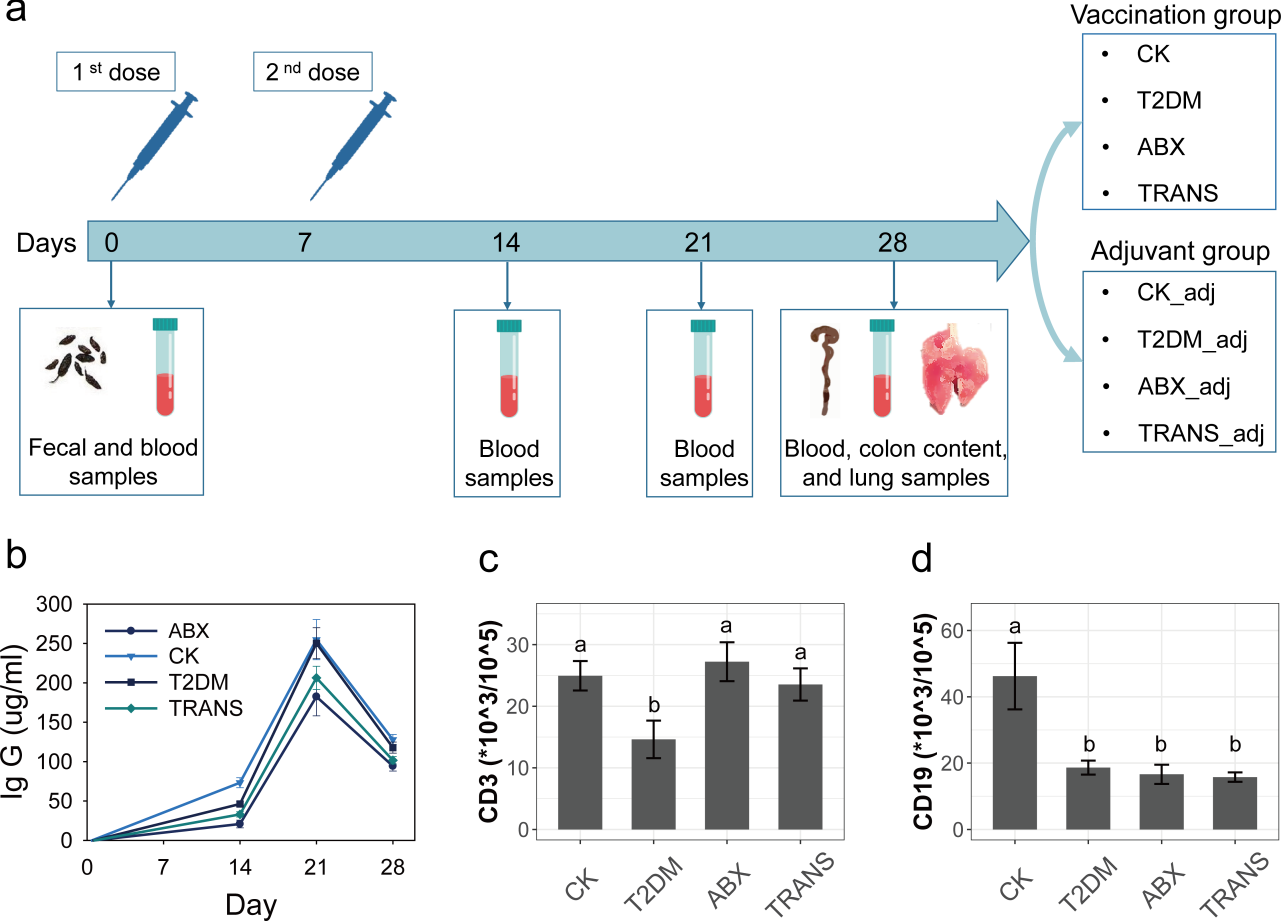
Sampling procedure and immune response. (**a**) Mice (CK = 6, T2DM = 11, ABX = 10, and TRANS = 11) received two intramuscular injections of inactivated vaccine with a 7-day interval. The mice (CK_adj = 4, T2DM_adj = 4, ABX_adj = 4, and TRANS_adj = 5) received the same amount of aluminum adjuvant as controls. Blood samples were collected before vaccination (day 0) and on days 14, 21, and 28. Fecal samples were collected on day 0, and colon content and lung samples were collected on day 28. (**b**) SARS-CoV-2-S1 specific IgG levels at days 0, 14, 21, and 28. (**c**) Frequency of blood cells positive for CD3. (**d**) Frequency of blood cells positive for CD19. Different letters in (c) and (d) stand for significant differences between groups (*P* < 0.05) by using Kruskal–Wallis tests.

Streptozotocin (STZ) injection was used to induce T2DM in mice, which were subsequently randomized into three groups: T2DM group (no intervention), ABX group (antibiotic cocktail treatment), and TRANS group (fecal microbiota transplantation from healthy mice) (Fig. 1). Healthy mice (CK group), the T2DM group, the ABX group, and the TRANS group received two doses of inactivated SARS-CoV-2 vaccine with a 7-day interval (Fig. 2a). The level of IgG antibody against the spike protein (IgG) was assessed using enzyme-linked immunosorbent assays (ELISAs) before vaccination and at 14, 21, and 28 days after the first dose.

All mice showed no detectable IgG at day 0 (Fig. 2b). T2DM significantly attenuated IgG levels compared with healthy mice (CK group) at day 14; however, the IgG levels of the T2DM group were comparable to those in the CK group at days 21 and 28 (Fig. S1). The ABX group demonstrated the lowest antibody levels among all groups on days 14, 21, and 28 (Fig. S1). Fecal microbiota transplantation significantly elevated the antibody levels compared to the ABX group at day 14, although they remained lower than those in the T2DM group (Fig. S1).

Flow cytometry was used to quantify cells staining positively for CD3 (T lymphocytes) and CD19 (B lymphocytes) in blood at day 28 (Fig. 2c and d). The T2DM group exhibited a decrease in the proportions of T lymphocytes and B lymphocytes compared to healthy mice. The ABX and TRANS groups showed a significant increase in the proportions of T lymphocytes compared to the T2DM group, with levels comparable to those of healthy mice.

### The vaccination influences the microbiota of the gut but not the lung

**Fig 3:**
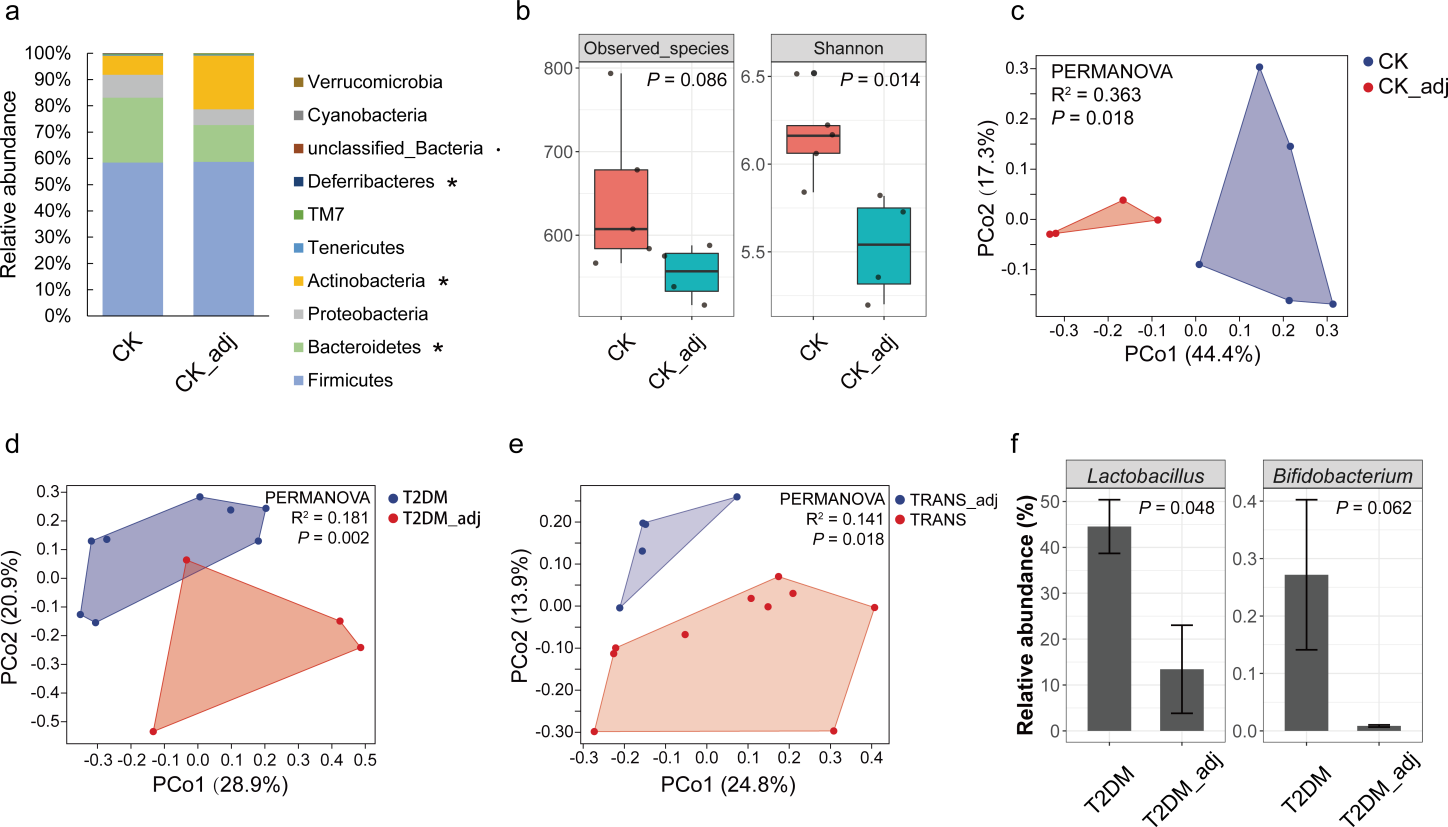
The impact of SARS-CoV-2 vaccination on gut microbiota. (**a**) Comparison of gut microbiota at the phylum level between CK and CK_adj groups by Wilcoxon rank sum tests. ^*^P < 0.05; ^•^*P* < 0.1. (**b**) Observed species and Shannon–Weiner index of gut microbiota in the CK group were significantly higher than in the CK_adj group, assessed using the Wilcoxon rank sum test. (**c**) PCoA and PERMANOVA analyses comparing beta diversity between CK and CK_adj groups. (**d**) PCoA and PERMANOVA analyses comparing beta diversity between T2DM and T2DM_adj groups. (**e**) PCoA and PERMANOVA analyses comparing beta diversity between TRANS and TRANS_adj groups. (**f**) Higher relative abundance of *Lactobacillus* and *Bifidobacterium* was observed in the T2DM group compared to the T2DM_adj group, as determined by Wilcoxon rank sum tests.

Colon contents were collected and subjected to 16S rRNA metagenomic sequencing on day 28. A total of 3,372,400 clean reads were obtained (Table S1), clustered into 10,413 amplicon sequence variants (ASVs). Rarefaction curves indicated a plateau, suggesting sufficient sequencing depth (Fig. S2). Comparisons between vaccinated CK mice (CK) and CK mice receiving adjuvant (CK_adj) were conducted to assess the vaccine’s impact on the gut microbiota. At the phylum level, significant differences in the relative abundance of Bacteroidetes, Actinobacteria, and Deferribacteres were observed between CK and CK_adj (Fig. 3a). Vaccination notably increased the evenness (Shannon–Weiner index) of the gut microbiota (Fig. 3b) and significantly influenced beta diversity, as determined by principal coordinate analysis (PCoA) and permutational multivariate analysis of variance (PERMANOVA) based on Bray–Curtis distance (Fig. 3c). At the genus level, vaccination resulted in significant increases in the relative abundance of *Bacteroides*, *Parabacteroides*, *Mucispirillum*, *Clostridium*, and *Ruminococcus*, while reducing the relative abundance of *Bifidobacterium* (Fig. S3). These findings highlight the significant impact of vaccination on the composition and structure of the gut microbiota in healthy mice.

Using PCoA and PERMANOVA, vaccination significantly altered the beta diversity of the gut microbiota in both T2DM and TRANS mice based on Bray–Curtis distance (Fig. 3d and e). At the genus level, vaccination significantly increased the relative abundance of *Lactobacillus* and *Bifidobacterium* in T2DM mice (Fig. 3f). These findings underscore the significant impact of vaccination on the beta diversity of the gut microbiota in both T2DM and TRANS mice.

To further investigate the impact of vaccination on lung microbiota, lung tissues were collected and subjected to 16S rRNA metagenomic sequencing on day 28. A total of 2,760,404 clean reads were obtained (Table S2), clustered into 3,919 ASVs. Rarefaction curves indicated adequate sequencing depth (Fig. S4). Analysis of alpha diversity, beta diversity, and the relative abundance of each phylum revealed no significant differences between CK and CK_adj (Tables S3 to S5), suggesting that vaccination had no discernible influence on the structure and composition of lung microbiota.

### The fecal microbiota before vaccination and the association with initial immune response

**Fig 4:**
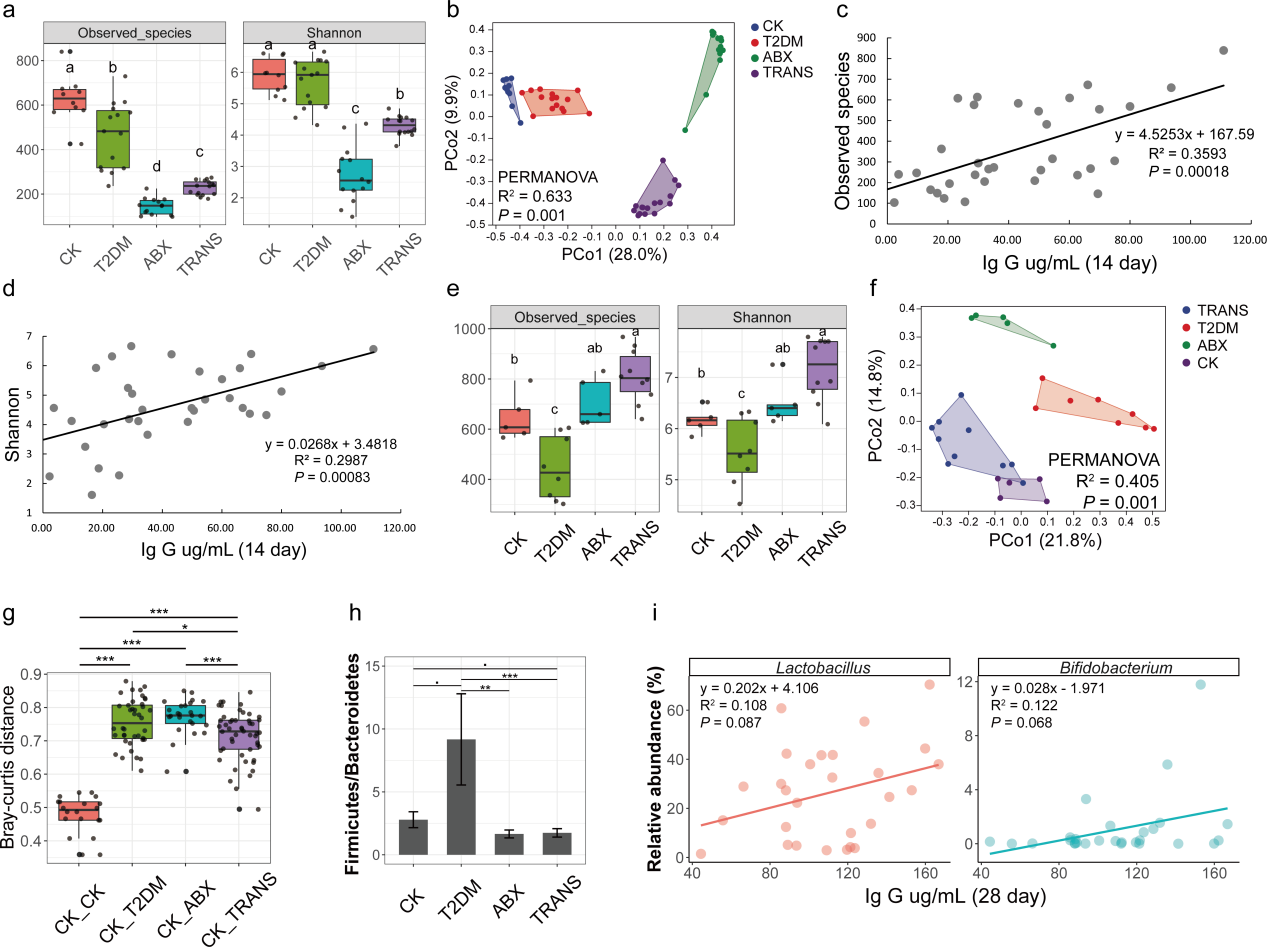
Variations of host microbiota and relationships with IgG level. (**a**) Alpha diversity of fecal microbiota (day 0) among the four groups assessed using Kruskal−Wallis tests. (**b**) PCoA and PERMANOVA analyses of fecal microbiota (day 0) among four groups. (**c**) Linear regression of observed species (day 0) and IgG levels (day 14). (**d**) Linear regression of Shannon−Weiner index (day 0) and IgG level (day 14). (**e**) Alpha diversity of gut microbiota (day 28) among the four groups assessed using Kruskal−Wallis tests. (**f**) PCoA and PERMANOVA analyses of gut microbiota (day 28) among the four groups. (**g**) Comparison of Bray−Curtis distance using Wilcoxon rank sum tests. CK_CK, distance within CK group; CK_T2DM, distance between CK group and T2DM group; CK_ABX, distance between CK group and ABX group; CK_TRANS, distance between CK group and TRANS group. (**h**) Comparison of Firmicutes/Bacteroidetes ratio among the four groups using Wilcoxon rank sum tests. (**i**) Linear regressions of the IgG level (day 28) and relative abundance of specific gut bacteria on day 28 (*Lactobacillus* and *Bifidobacterium*). Different letters represent significant differences (*P* < 0.05). ^***^P < 0.001; ^**^P < 0.01; ^*^P < 0.05; ^•^*P* < 0.1.

Fecal samples collected at day 0 were subjected to 16S rRNA metagenomic sequencing. A total of 4,642,043 clean reads were obtained (Table S1), clustered into 7,207 ASVs. Rarefaction curves indicated sufficient sequencing depth (Fig. S2). Analysis revealed significant changes in alpha and beta diversity of fecal microbiota among the four groups (Fig. 4a and b), indicating that T2DM disrupted the composition and structure of fecal microbiota in healthy mice, and FMT altered the composition of fecal microbiota in mice with T2DM.

Further analysis examined the relationship between fecal microbiota and immune response. Spearman’s correlation analyses revealed positive correlations between alpha diversity and the IgG levels on day 14 (observed species: *r* = 0.56, *P* = 0.0005; Shannon–Weiner: *r* = 0.53, *P* = 0.0014), which were confirmed by linear regression analyses (Fig. 4c and d). Specific genera, including *Desulfovibrio*, *Oscillospira*, *Adlercreutzia*, *Ruminococcus*, and *Allobaculum*, were positively correlated with the IgG level on day 14 (Table S6). These findings suggested a potential relationship between the composition of the fecal microbiota and the immune response.

### Host microbiota and the association with immune response after 28 days of vaccination

The alpha and beta diversity of gut microbiota on day 28 varied significantly among the four groups (Fig. 4e and f). Furthermore, the Bray–Curtis distance between the CK and TRANS groups was significantly smaller compared to that between the CK and T2DM (Fig. 4g). The Firmicutes/Bacteroidetes ratio (F/B ratio) in the T2DM group was significantly higher than that in the CK, ABX, and TRANS groups (Fig. 4h). These findings indicated that T2DM significantly impacted the gut microbiota of vaccinated mice, while FMT significantly reduced the differences in gut microbiota between T2DM and CK mice.

To delve into the relationship between immune response and gut microbiota, Spearman’s correlation analyses were employed. Among the top 30 families, the relative abundance of Desulfovibrionaceae and Prevotellaceae exhibited negative relationships with the level of IgG on day 28 (Fig. S5). Among the top 100 genera, the relative abundances of *Bifidobacterium* and *Lactobacillus* positively correlated with the level of IgG on day 28 (Fig. 4i). Conversely, the relative abundances of *Prevotella*, *Butyricimonas*, *Desulfovibrio*, *Alistipes*, *Staphylococcus*, and *Dorea* showed negative correlations (Fig. S6a). Except for *Desulfovibrio*, the relative abundance of these genera significantly varied among the four groups (Fig. S6b).

Moreover, the evenness of lung microbiota exhibited a positive relationship with the IgG levels on day 28 (Fig. S7). At the family level, Bacillaceae, Comamonadaceae, Halomonadaceae, and Pseudonocardiaceae (lung microbes) were significantly positively correlated with the IgG levels on day 28 based on Spearman’s correlation analyses (Fig. S8).

### The potential relationships between immune response and gut metabolome

**Fig 5:**
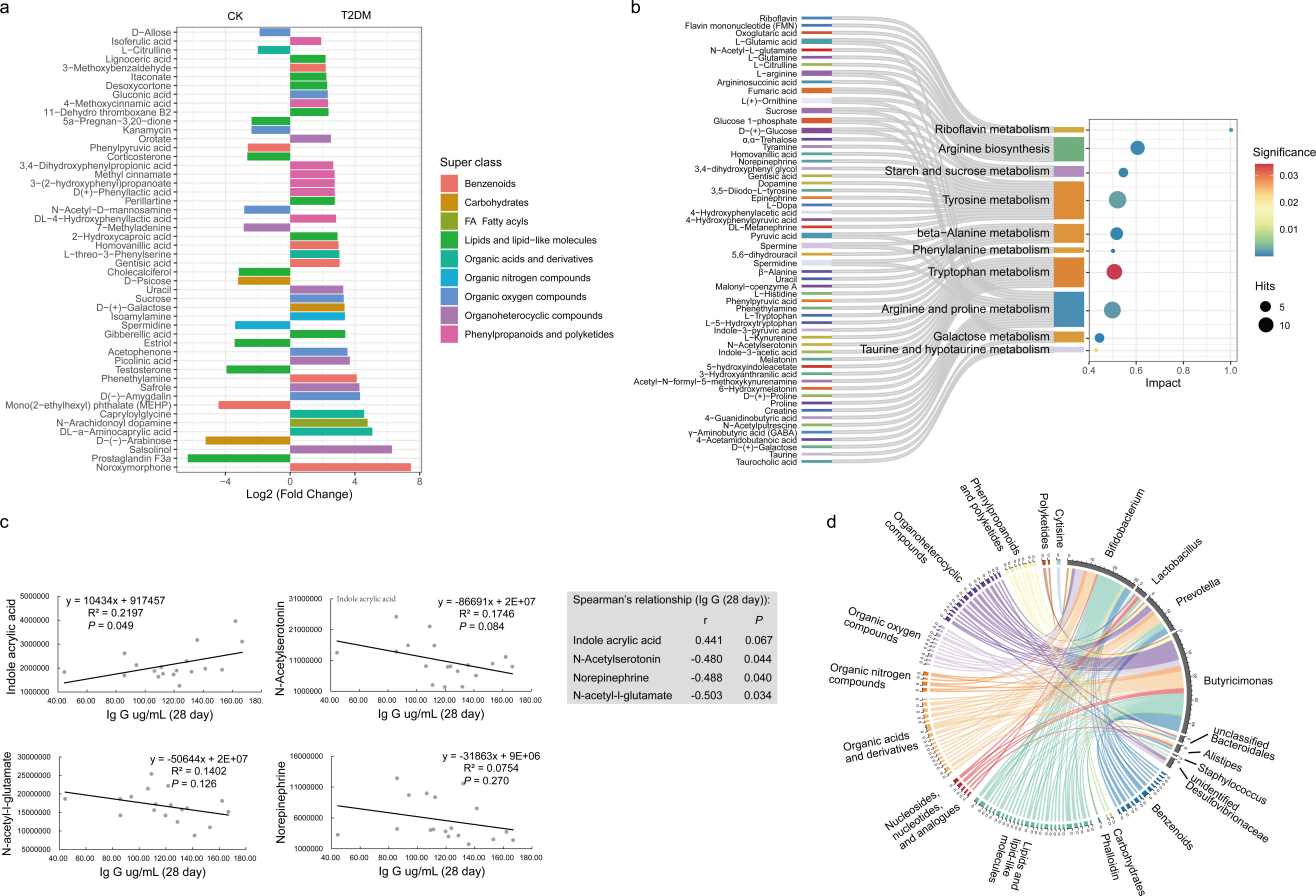
Variation in gut metabolome and its relationship with IgG levels. (**a**) Top 50 significantly altered metabolites between CK and T2DM groups. (**b**) Top 10 significantly changed pathways between CK and T2DM groups. (**c**) Relationships between specific gut metabolites (indole acrylic acid, N-acetylserotonin, norepinephrine, and N-acetyl-l-glutamate) and IgG levels (day 28) assessed using linear regression and Spearman’s correlation. (**d**) Circos plot depicting Spearman’s relationships (|*r*| > 0.7, *P* < 0.05) between metabolites and specific gut bacteria (day 28), which exhibited significantly altered relative abundance among the four groups and were associated with IgG levels on day 28.

It has been well established that the gut microbiota participates in host regulation via the modulation of gut metabolites (28). Therefore, we conducted an untargeted metabolic analysis to investigate variations in gut metabolites on day 28 among three groups (CK, T2DM, and TRANS). A significant variation in the composition of gut metabolites was observed among the three groups (Fig. S9a; analysis of similarity: *R* = 0.4424, *P* = 0.004). A total of 735 metabolites were significantly different (*P* < 0.05 and |Log2(fold change)| > 1) between the CK and T2DM groups (Fig. S9b), while 279 metabolites exhibited significant differences between the TRANS and T2DM groups (Fig. S9c). Levels of prostaglandin F3α, mono(2-ethylhexyl) phthalate, D-psicose, 7-methyladenine, and N-acetyl-D-mannosamine were higher in the CK and TRANS groups compared to the T2DM group. Conversely, the levels of D-(+)-galactose, sucrose, uracil, homovanillic acid, DL-4-hydroxyphenyllactic acid, D(+)-phenyllactic acid, 3-(2-hydroxyphenyl)propanoate, methyl cinnamate, 3,4-dihydroxyphenylpropionic acid, and desoxycortone were significantly higher in the T2DM group compared to the CK and TRANS groups (Fig. 5a; Fig. S10a).

Metabolic pathway enrichment analyses of the differential metabolites were conducted using the KEGG database. Metabolic pathways with *P*-value <0.05 were considered significantly enriched with differential metabolites, and the top 10 pathways with the greatest impact were depicted as bubble charts (Fig. 5b; Fig. S10b). The top 10 significantly altered pathways between CK and T2DM were riboflavin metabolism, arginine biosynthesis, starch and sucrose metabolism, tyrosine metabolism, beta-alanine metabolism, phenylalanine metabolism, tryptophan metabolism, arginine and proline metabolism, galactose metabolism, and taurine and hypotaurine metabolism (Fig. 5b). In comparison to T2DM, tyrosine metabolism, galactose metabolism, starch and sucrose metabolism, beta-alanine metabolism, and beta-alanine metabolism showed variation in both CK and TRANS (Fig. 5b; Fig. S10b).

The relationships between immune response and gut metabolites were analyzed by using Spearman’s correlation and linear regression. The IgG antibody level on day 28 was positively correlated with the level of indole acrylic acid, a tryptophan metabolite (Fig. 5c). Conversely, the IgG level on day 28 exhibited negative relationships with N-acetylserotonin (a tryptophan metabolite), norepinephrine (a tyrosine metabolite), and N-acetyl-l-glutamate, involved in the synthesis of L-arginine (Fig. 5c). Additionally, the level of indole acrylic acid showed significant positive relationships with the relative abundance of *Lactobacillus*, *Allobaculum*, and *Clostridium* (gut bacterium on day 28) and negative relationships with *Ruminococcus*, *Butyricimonas*, *Vagococcus*, *Corynebacterium*, *Prevotella*, and *Enterococcus* (gut bacteria on day 28; Table S7).

To further explore the relationships between gut metabolites and microbiota, we utilized a Circos plot to visualize Spearman’s correlations (|*r*| > 0.7, *P* < 0.05) between metabolites and specific gut bacteria, which exhibited significantly altered relative abundances among the four groups and were associated with the level of IgG on day 28 (Fig. 5d). *Bifidobacterium* showed correlations with organic oxygen compounds, organic acids and derivatives, lipids and lipid-like molecules, and benzenoids. *Lactobacillus* exhibited correlations with nucleosides, nucleotides, analogs, lipids, and lipid-like molecules. *Prevotella* demonstrated close associations with organoheterocyclic compounds, organic acids and derivatives, lipids and lipid-like molecules, and benzenoids. *Butyricimonas* displayed close relationships with phenylpropanoids and polyketides, organoheterocyclic compounds, organic oxygen compounds, organic nitrogen compounds, organic acids and derivatives, nucleosides, nucleotides, analogs, lipids and lipid-like molecules, and benzenoids.

## DISCUSSION

This study, employing a mouse model, investigated the impact of T2DM on the efficacy of SARS-CoV-2 vaccination and elucidated the interplay among T2DM, vaccine efficacy, and host microbiota (gut and lung). The findings revealed that T2DM significantly attenuated the initial IgG antibody response to the SARS-CoV-2 vaccine, aligning with prior reports indicating impaired antibody responses in T2DM patients post-vaccination (4, 29). However, this suppression effect dissipated within 21 days, with both healthy and T2DM mice reaching peak IgG antibody levels, indicating comparable protection provided by two doses of the vaccine. Previous investigations have also underscored the efficacy of full SARS-CoV-2 vaccination (including inactivated virus vaccine, mRNA vaccine, recombinant protein vaccine, and viral vector vaccine) or two doses across various vaccine types for T2DM patients (30–32). Nonetheless, Cheng et al. observed diminished antibody responses to two doses of inactivated SARS-CoV-2 vaccine in diabetic individuals, particularly those with poor glycemic control (29). Thus, optimizing the vaccination schedule for T2DM patients remains imperative to enhance the protective efficacy of SARS-CoV-2 vaccines.

Germ-free mice have impaired antibody responses to the model antigen ovalbumin (33). Additionally, the Bacillus Calmette–Guerin (BCG) vaccine has shown impaired antigen-specific IgG responses in mice treated with antibiotics (34). Moreover, the administration of antibiotics within 6 months before vaccination has been associated with gut dysbiosis and lower seroconversion rates in recipients of the BNT162b2 vaccine (35). Consistent with these findings, our study observed significantly lower IgG levels in the ABX group compared to the T2DM and CK groups, along with a corresponding decrease in alpha diversity of the gut microbiota. Therefore, the impaired antibody responses observed in the ABX group may be attributable to antibiotic-induced gut microbiota dysbiosis.

The significant elevation in IgG levels observed in TRANS mice compared with ABX mice after 14 days of the first dose indicates the potential of FMT to rescue impaired immune responses, as previously reported for BCG vaccines (34). However, despite this improvement, the IgG levels in the TRANS group remained lower than those in the T2DM group at day 14. Interestingly, before vaccination, the alpha diversity (richness and evenness) of fecal microbiota exhibited a positive relationship with IgG levels at day 14, with a decline in alpha diversity corresponding to decreasing IgG levels (CK > T2DM >TRANS > ABX). Similar associations between the composition of gut microbiota and vaccine responses have been reported in recent studies on clinical cohorts and animals (36). Specifically, Daddi et al. discovered that Shannon diversity and richness of the gut microbiota before SARS-CoV-2 mRNA vaccination were significantly positively correlated with IgG levels (19). Therefore, we suspected that the impaired immune responses of TRANS mice may be correlated with the alpha diversity of fecal microbiota before vaccination. Additionally, a higher relative abundance of Actinobacteria was observed in the CK group before vaccination than in the T2DM, ABX, and TRANS groups, consistent with previous studies linking higher Actinobacteria abundance to greater vaccine responses (37). Thus, we suspected that the initial IgG responses of mice with T2DM may be related to the pre-vaccination signatures of gut microbiota, including alpha diversity and Actinobacteria abundance.

FMT, a promising therapeutic strategy for T2DM patients, exhibits varying efficacy depending on donor and recipient conditions and laboratory settings (38). In this study, the partial effectiveness of FMT may be attributed to factors such as the microbial load of the recipient, stool processing procedures, and duration of FMT (38). Recipients with a low microbial load tend to show more effective FMT outcomes compared to those with a high microbial load (39). Extending the duration of the antibiotic regimen may help deplete the gut microbiota of recipients more thoroughly. In a previous study, an 8-week daily FMT in a T2DM mouse model successfully reversed insulin resistance and impaired islets (24). A proper extension of the duration of FMT may improve its effectiveness. The gut microbiota consists of an abundance of obligate anaerobic bacteria (40). Collecting stool samples in ambient air can reduce the abundance of various microbes (40). To mitigate the influence of oxygen, stool samples can be preserved in maltodextrin, trehalose, or a medium containing antioxidants (41), which could improve the effectiveness of FMT in future research.

Although no significant relationship was found between alpha diversity (day 28) and IgG level (day 28), the relative abundances of *Bifidobacterium* and *Lactobacillus* showed positive correlations with the IgG level (day 28). *Bifidobacterium* and *Lactobacillus* are well known for their probiotic activities, which include enhancing vaccine effectiveness. Oral consumption of species belonging to *Bifidobacterium* and *Lactobacillus* has been shown to confer immune benefits for both intramuscularly injected influenza vaccines and oral cholera vaccines (42–44). Moreover, fecal *Bifidobacterium* of early infancy was positively associated with tetanus toxoid-specific IgG levels after vaccination (45). After SARS-CoV-2 vaccination, a higher abundance of *Bifidobacterium adolescentis* was observed in subjects with high neutralizing antibodies (46). These studies provide evidence supporting the idea of targeting specific gut probiotics to enhance vaccine efficacy. Therefore, we speculated that gut probiotics, *Bifidobacterium* and *Lactobacillus*, may modulate the immune response to the SARS-CoV-2 vaccine, although further validation through animal experiments with specific microbial intervention is needed.

The role of gut microbiota in immune responses to SARS-CoV-2 vaccination remains to be fully elucidated. However, previous studies have proposed several potential mechanisms linking gut microbiota to vaccine efficacy (47). Firstly, gut microbiota can modulate systemic immune response through microbial metabolites, such as SCFAs, tryptophan metabolites, and secondary bile acids (47). For instance, SCFAs may enhance antibody responses to vaccination by improving B cell metabolism, plasma cell differentiation, and class switching (48). Additionally, the gut microbiota serves as a constant source of natural adjuvants, enhancing immune responses to vaccines through the stimulation of immune cells (49). Moreover, some gut microbes possess antigens similar to vaccines, thereby enhancing immune responses through cross-reactivity (47). Jia et al. demonstrated that gut microbial proteins, such as *Escherichia coli*-derived heat shock protein 60 and 70, which bear similarity to the linker domain of the SARS-CoV-2 protein S2, could potentially enhance the immune response to SARS-CoV-2 vaccine (47, 50).

The administration of the SARS-CoV-2 vaccine induced significant alterations in the composition and structure of the gut microbiota in both healthy mice and those with T2DM, while exhibiting negligible effects on lung microbiota. This is consistent with previous observations suggesting a greater influence of the BCG vaccine on gut microbiota compared to lung microbiota (51). Similar effects on the gut microbiota have been observed with other vaccines, including the inactivated bivalent *Aeromonas hydrophila*/*Aeromonas veronii* vaccine and the BBIBP-CorV vaccine (51–53). However, inconsistent opinions have been proposed regarding the stability of the gut microbiota post-vaccination (54). In this study, when all vaccinated mice were amalgamated into a single group (referred to as “immune”) and contrasted with mice receiving adjuvant (termed “adjuvant”), the beta diversity showed no significant difference between the two groups by using PCoA and PERMANOVA analysis (Fig. S11a; PERMANOVA: *P* = 0.117). The alpha diversity also exhibited no significant difference between the immune and adjuvant groups (Fig. S11b). Therefore, we suspected that the inconsistency of results among different studies may be due to differences in the composition and structure of the gut microbiota and the underlying disease of individuals before vaccination. Furthermore, the relative abundance of *Lactobacillus* and *Bifidobacterium* exhibited different tendencies among CK, T2DM, and TRANS mice. Only mice with T2DM showed an elevated relative abundance of *Lactobacillus* and *Bifidobacterium* after vaccination. This result further suggests that the composition and structure of gut microbiota and the underlying disease of individuals may be influencing factors for the effect of vaccination on gut microbiota.

Gut metabolites have been shown to play regulatory roles in the gut, affecting the host’s immune system and overall health status (55) and influencing the effectiveness of vaccines (10). In this study, significant variations were observed in gut riboflavin metabolism, arginine biosynthesis, and tyrosine and tryptophan metabolism between CK and T2DM, between which the difference in IgG levels (days 14 and 28) was detected. These pathways have been reported to be involved in the regulation of host immunity. Riboflavin is associated with the activation of mucosal-associated invariant T cells to produce inflammatory cytokines (56). Arginine depletion depresses T cell proliferation, while replenishment of arginine maximizes proliferation (57, 58). Gut microbiota with enhanced tyrosine metabolism reduced CCL20 production by airway epithelial cells (59). Dysbiosis in tryptophan metabolism is associated with inflammatory diseases (60). Moreover, the metabolisms of tryptophan and tyrosine were enriched in recipients of the inactivated influenza vaccine (61).

Several gut metabolites are related to IgG levels on day 28, including indole acrylic acid (bacterial tryptophan metabolite), N-acetylserotonin (endogenous tryptophan metabolite), norepinephrine (tyrosine metabolite), and N-acetyl-l-glutamate [involved in the synthesis of L-arginine (62)], among which indole acrylic acid showed a positive correlation. Previous research has suggested a potential therapeutic strategy of increasing intestinal indole acrylic acid production in patients with inflammatory bowel disease and rats with T2DM by improving the integrity of the intestinal barrier and mitigating inflammatory responses (63–65). Therefore, we suspected that tryptophan metabolite (indole acrylic acid) played a potential role in regulating SARS-CoV-2 vaccine effectiveness. Future research needs to explore the relationship between characteristic gut metabolites and vaccine efficacy to improve the immune efficacy of SARS-CoV-2 vaccines.

In conclusion, our study found that the SARS-CoV-2 inactivated vaccine significantly altered the composition of gut microbiota but not lung microbiota. Initially, T2DM impaired the IgG response to the vaccine, which was restored to the level of healthy mice after 28 days. The initial IgG response positively correlated with the alpha diversity of fecal microbiota before vaccination. By day 28 post-vaccination, the relative abundance of gut microbes, *Bifidobacterium*, and *Lactobacillus*, as well as the level of gut bacterial tryptophan metabolite (indole acrylic acid), showed positive relationships with the IgG level. Furthermore, the level of indole acrylic acid positively correlated with the relative abundance of *Lactobacillus*. These findings suggest that targeting the gut microbiota of T2DM individuals could potentially enhance the efficacy of SARS-CoV-2 inactivated vaccines. Strategies involving probiotics, prebiotics, FMT, or dietary interventions may offer promising avenues for improving vaccine outcomes in this population. Our results may provide new insights into the mechanisms of SARS-CoV-2 vaccine efficacy in T2DM patients and suggest potential clinical applications. Nonetheless, further experimental studies are essential to establish causal relationships between vaccine efficacy in T2DM individuals and gut microbiota through more refined FMT and targeted interventions. Moreover, future research exploring the molecular pathways linking gut microbiota and the immune system’s response to SARS-CoV-2 vaccines is crucial for a deeper understanding of how gut microbiota modulation can optimize protective vaccine responses in T2DM individuals.

## MATERIALS AND METHODS

### Animal

Four-week-old male C57BL/6 mice were obtained from the Laboratory Animal Department of Hubei University of Medicine. They were housed in polypropylene cages (three to five per cage) with *ad libitum* access to food and water. The ambient conditions maintained a room temperature of 22°C ± 2°C, with a relative humidity of 60% ± 5%, under a 12-hour light/dark cycle. The ambient conditions remained stable and consistent throughout the animal experiments.

### Diabetes modeling

Male C57BL/6 mice were assigned to different dietary groups: a standard diet group (CK group, 12.7% fat, 64.3% carbohydrate, and 23.0% protein; Jiangsu Xietong Pharmaceutical Bio-engineering Co., Ltd., Jiangsu, China) and a high-fat diet group (HFD group, 60% fat, 20% carbohydrate, and 20% protein, D12492 diet; Jiangsu Xietong Pharmaceutical Bio-engineering Co., Ltd., Jiangsu, China). Mice were provided with sterile water and food *ad libitum* for 6 weeks. Subsequently, mice in the HFD group received intraperitoneal injections of STZ (40 mg/kg; Sigma-Aldrich, St Louis, MO, USA) for 5 consecutive days, which led to partial pancreatic β-cell damage, corresponding to a type 2 diabetes-like phenotype according to previous reports (66, 67). The mice in the normal control group were intraperitoneally injected with citrate–phosphate buffer. All mice were fasted for 6–8 hours prior to STZ treatment. All mice were fed with the standard diet till the end of the experiments. Blood glucose levels were measured 1 week after the final injection following an overnight fasting. Mice with blood glucose levels exceeding 11.1 mmol/L (above 200 mg/dL) were considered to be type 2 diabetic mice (Fig. S12) (68).

### Transplantation of fecal microbiota

The diabetic mice were randomly divided into three groups: the T2DM group, the ABX group, and the TRANS group (Fig. 1). The gut microbiota of the ABX and TRANS groups were depleted by using antibiotics, as previously described (38, 69). A broad-spectrum antibiotic cocktail regime (ampicillin 1  g/L, neomycin sulfate 1  g/L, metronidazole 1  g/L, and vancomycin 0.5 g/L; Sigma-Aldrich Co. Ltd, Missouri, USA) was administered in the drinking water *ad libitum* to the TRANS group for 5 consecutive days and to the ABX group for 15 consecutive days. The drinking solution was renewed daily. Then, the ABX and TRANS groups were provided with normal autoclaving water till the end of the experiments. The CK and T2DM groups were provided *ad libitum* access to normal autoclaving water.

Post-antibiotic washout, the recipients (TRANS group) received donor microbiota (CK group) via oral gavage. CK mice were placed in a cage sterilized with 75% ethanol and lined with sterilized filter paper. Fecal samples were promptly collected after defecation and stored in a 4°C freezer until transplantation (within 4 hours). Fecal microbiota was prepared by diluting a 300- mg fecal sample homogenized in 1 mL of sterile phosphate-buffered saline (PBS). The suspension was centrifuged for 30 s at 300 g, and 0.2 mL of the supernatant was administered via gavage to each recipient every other day for 10 days (Fig. 1).

### Animal immunization and sampling strategy

At day 0, fecal and blood samples were collected from CK, T2DM, ABX, and TRANS mice. Fecal samples were immediately frozen in liquid nitrogen and stored at −80°C until use. Blood samples were allowed to clot for 30 minutes, then centrifuged (3,000 × *g*, 10 minutes, 22°C), and serum was collected and stored at −80°C until analysis. Subsequently, mice (CK = 6, T2DM = 11, ABX = 10, and TRANS = 11) received two intramuscular injections (200 µL, 3 µg/0.5 mL) of inactivated vaccine (Sinovac Biotech Ltd., Beijing, China) with each hind limb receiving 100 µL, with a 7-day interval (70). The mice (CK_adj = 4, T2DM_adj = 4, ABX_adj = 4, and TRANS_adj = 5) received the same amount of aluminum adjuvant as controls.

Blood samples were collected on days 14 and 21, and serum samples were collected and stored as previously described. Upon sacrifice, blood samples, colon contents, and whole lung tissues were harvested on day 28 (Fig. 2).

### Enzyme-linked immunosorbent assay

For the determination of SARS-CoV-2-S1 specific IgG level, ELISAs were performed following established protocols (70, 71). Briefly, 96-well plates (701001, Wuxi NEST Biotechnology Co., Ltd., Jiangsu, China) were coated with 0.1 µg of purified S1 protein (SC1806P, GenScript Biotech Corporation, Piscataway, NJ, USA) overnight at 2–8°C, then blocked with 2% bovine serum albumin for 1 hour at room temperature. Diluted sera (1:100) were applied to each well for 2 hours at 37°C, followed by incubation with goat anti-mouse antibodies (A0216, Beyotime, Shanghai, China) conjugated with horseradish peroxidase for 1 hour at 37°C after three times of PBS wash. The plate was developed using 3,3′5,5′-tetramethylbenzidine, following 2 M H_2_SO_4_ addition to stop the reaction, and read at 450/630 nm by an ELISA plate reader for final data.

### Flow cytometry analysis for humoral immune responses

Whole blood samples from day 28 were analyzed using a CytoFLEX benchtop flow cytometer (Beckman Coulter Life Sciences, Indianapolis, IN, USA) with anti-CD3 and anti-CD19 fluorochrome-conjugated monoclonal antibodies (CD3: 300331, BioLegend; CD19: 11-0199-41, eBioscience) (72). The stained blood samples were lysed with a diluted lysing solution, and special care was taken not to expose the stained sample to light. CD3^+^ T cells were identified following published protocols (73). B cells were identified by CD19 expression. Results were expressed as the percentage of mononuclear cells that stained positively.

### 16S rRNA metagenomic sequencing

Feces, colon contents, and whole lung tissue were promptly submerged in liquid nitrogen and stored at −80°C until DNA extraction. Total DNA was extracted using a Power Soil DNA isolation kit (MO Bio, USA) following the manufacturer’s instructions. DNA integrity was verified via 1% agarose gel electrophoresis, and DNA concentration was determined using NanoDrop NC2000 (Thermo Scientific). For sequencing, PCR amplification of the bacterial 16S rRNA gene V4 region was performed using the forward primer F515 (5ʹ-GTGYCAGCMGCCGCGGTAA-3ʹ) and the reverse primer R806 (5ʹ-GGACTACNVGGGTWTCTAAT-3ʹ) (74). Sample-specific 7 bp barcodes were incorporated into the primers for multiplex sequencing. The PCR components contained 5 µL of buffer (5×), 0.25 µL of Fast pfu DNA Polymerase (5 U/µL), 2 µL (2.5 mM) of dNTPs, 1 µL (10 µM) of each forward and reverse primer, 1 µL of DNA template, and 14.75 µL of ddH_2_O. Thermal cycling consisted of initial denaturation at 98°C for 5 minutes, followed by 25 cycles consisting of denaturation at 98°C for 30 s, annealing at 53°C for 30 s, and extension at 72°C for 45 s, with a final extension of 5 minutes at 72°C. Purified and qualified amplicons were pooled in equal amounts, and sequencing libraries were generated using the TruSeq DNA PCR-Free Sample Preparation Kit (Illumina, USA). The libraries were then sequenced on an Illumina NovaSeq-PE250 platform at Shanghai Personal Biotechnology Co., Ltd. (Shanghai, China).

### Metabolome analysis

The colon content sample (25 mg) was extracted with 800 µL of extracting solution (methanol:acetonitrile:water = 2:2:1, vol:vol:vol) containing 10 µL of internal standard. The mixture was then centrifuged at 25,000 rpm for 15 minutes at 4°C. After lyophilizing the supernatant, it was redissolved with 600 µL of complex solution (methanol:water = 1:9, vol:vol), followed by another centrifugation at 25,000 rpm for 15 min at 4°C. A 50-µL aliquot of the supernatant from each sample was taken and mixed with synthetic quality control samples to evaluate the repeatability and stability of the analysis process.

Ultra-high-performance liquid chromatography-mass spectrometry (UPLC–MS) analyses were conducted using Waters UPLC I-Class Plus (Waters, USA)–tandem Q Exactive high-resolution mass spectrometer (Thermo Fisher Scientific, USA) for metabolite separation and detection at BGI (Shenzhen, China). The offline mass spectrometry data were imported into Compound Discoverer 3.2 software (Thermo Fisher Scientific, USA) and analyzed in combination with the BGI metabolome database, mzCloud database, and ChemSpider online database. This analysis generated a data matrix containing information such as metabolite peak area and identification results (75).

### Statistical methods

Microbiome bioinformatics was conducted using QIIME2 2019.4 (https://docs.qiime2.org/2019.4/tutorials/), following the official tutorial with minor adjustments based on a previous study (76). Raw sequence data underwent demultiplexing with the Demux plugin, followed by primer trimming using the Cutadapt plugin (77). Reads were then filtered, denoised, and merged, and chimeric sequences were removed using the DADA2 plugin (78). Feature sequences and ASV tables were combined, and singleton ASVs were removed. Non-singleton ASVs were aligned using MAFFT (79) and used to construct a phylogenetic tree with FastTree (80). Taxonomic identification was performed using the classify-sklearn naïve Bayes taxonomy classifier, utilizing the SILVA database (Release 132). ASV abundance information was normalized based on a standard of sequence numbers corresponding to 95% of the sample with the least sequences. Subsequent analyses were conducted on normalized data.

QIIME2 and the R packages (v4.0.4) were used for sequence data analysis. The comparisons of IgG, CD3 and CD19 levels, alpha diversity, and relative abundance of specific microbes among four groups (CK, T2DM, ABX, and TRANS) were conducted using Kruskal–Wallis tests, employing the R package “Agricolae 1.3-5” (81). Observed species and Shannon diversity index were computed using the ASV table in QIIME2. ASV-level rarefaction curves were generated. The comparisons of alpha diversity and the relative abundance of specific microbes between two groups were conducted using Wilcoxon rank sum tests, employing the R package “stats 4.3.2.” PCoA, PERMANOVA, and analyses of similarities (ANOSIM) based on Bray–Curtis distance were implemented by using the R packages “ape 5.5” (82) and “vegan 2.5-7” (83). Linear regression and Spearman’s correlation analyses were employed to explore the relationships between microbiota features (alpha diversity and specific microbe abundance) and vaccine responses, using the R package “stats 4.3.2” and “Hmisc 5.1-1,” respectively (84).

Principal component analysis (PCA) and ANOSIM were employed to analyze the differences in the gut metabolome among the CK, T2DM, and TRANS groups. PCA was conducted using the R package “ade4 1.7-18” (85). To analyze the variation of gut metabolites, the value of variable importance in the projection (VIP) of the first principal component in orthogonal projections to latent structures discriminant analysis was obtained using SIMCA 16.0.2 software. Gut metabolites with VIP >1 and *P* < 0.05 were considered significantly varied between different groups (86). MetaboAnalyst 5.0 was used to analyze the metabolic pathways based on the differential metabolites, and the pathways were considered significant when *P* < 0.05 (87). Circos plot was generated using R package “circlize 0.4.15” (88). Box plots, dot plots, bubble diagrams, and histograms were generated using the R package “ggplot2 3.3.5” (89).

## Data Availability

The raw metagenomic sequencing data generated in this study have been deposited in GenBank under small read archive (SRA) in BioProject: PRJNA1106354 and PRJNA1105891.

## AFTER EPUB

*[This article was published on 27 August 2024 with Gong Cheng's name rendered as Cheng Gong and errors in the affiliations. The byline was corrected on 29 August 2024, and the affiliations were corrected in the current version, posted on 5 September 2024.]*

## SUPPLEMENTAL MATERIAL

Supplemental figures msphere.00380-24-s0001.pdf Figures S1 to S12.

Supplemental tables msphere.00380-24-s0002.pdf Tables S1 to S7.

ASM does not own the copyrights to Supplemental Material that may be linked to, or accessed through, an article. The authors have granted ASM a non-exclusive, world-wide license to publish the Supplemental Material files. Please contact the corresponding author directly for reuse.
