# Supplementary material for: Exploring the associations between gut microbiota composition and SARS-CoV-2 inactivated vaccine response in mice with type 2 diabetes mellitus
Source: mSphere. 2024 Aug 27;9(9):e00380-24. doi: 10.1128/msphere.00380-24 (PMC11423585; doi:10.1128/msphere.00380-24)
Supplement: Supplemental figures — Figures S1 to S12. [file msphere.00380-24-s0001.pdf]

Exploring the associations between gut microbiota composition and SARS-CoV-2 inactivated vaccine response in mice with type 2 diabetes mellitus

Long Liu[#1, 3, 4], Xianzhen He[#1, 2], Jiaqi Wang[1], Moran Li[1], Xiuli Wei[1], Jing Yang[1], Gong Cheng[5], Weixing Du*[1, 3], Zhixin Liu*[1, 3, 4], Xiao Xiao*[1, 3, 4]

[1] Department of Pathogen Biology, School of Basic Medical Sciences, Hubei University of Medicine, Shiyan, China; Department of Infectious Diseases, Renmin Hospital, Hubei University of Medicine, Shiyan, Hubei 442000, PR China

[2] Department of Children's Medical Center, Renmin Hospital, Hubei University of Medicine, Shiyan, China

[3] Institute of Virology, Shiyan Key Laboratory of Virology, Hubei University of Medicine, Shiyan, China

[4] Hubei Key Laboratory of Embryonic Stem Cell Research, Hubei University of Medicine, Shiyan, China

[5] New Cornerstone Science Laboratory, Tsinghua-Peking Joint Center for Life Sciences, School of Basic Medical Sciences, Tsinghua University, Beijing, China

# These authors contributed equally to this article.

* Corresponding authors.

Xiao Xiao, xiao910512@outlook.com

Zhixin Liu, lzx20022456@126.com

Weixing Du, duwx-025@163.com

Running title: Gut microbiota and SARS-CoV-2 vaccine response in mice

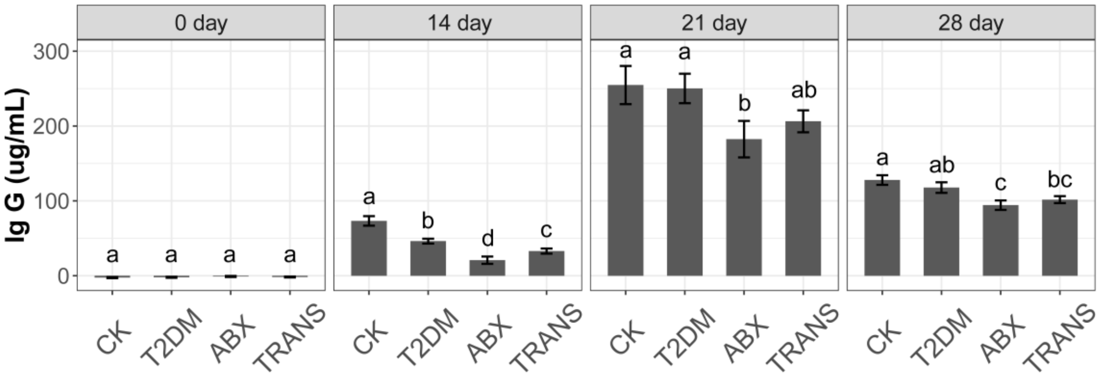

Figure S1. The comparison of the level Ig G between four groups (CK, T2DM, ABX and TRANS). Different alphabets stand for significant differences ($P < 0.05$) using Kruskal–Wallis tests.

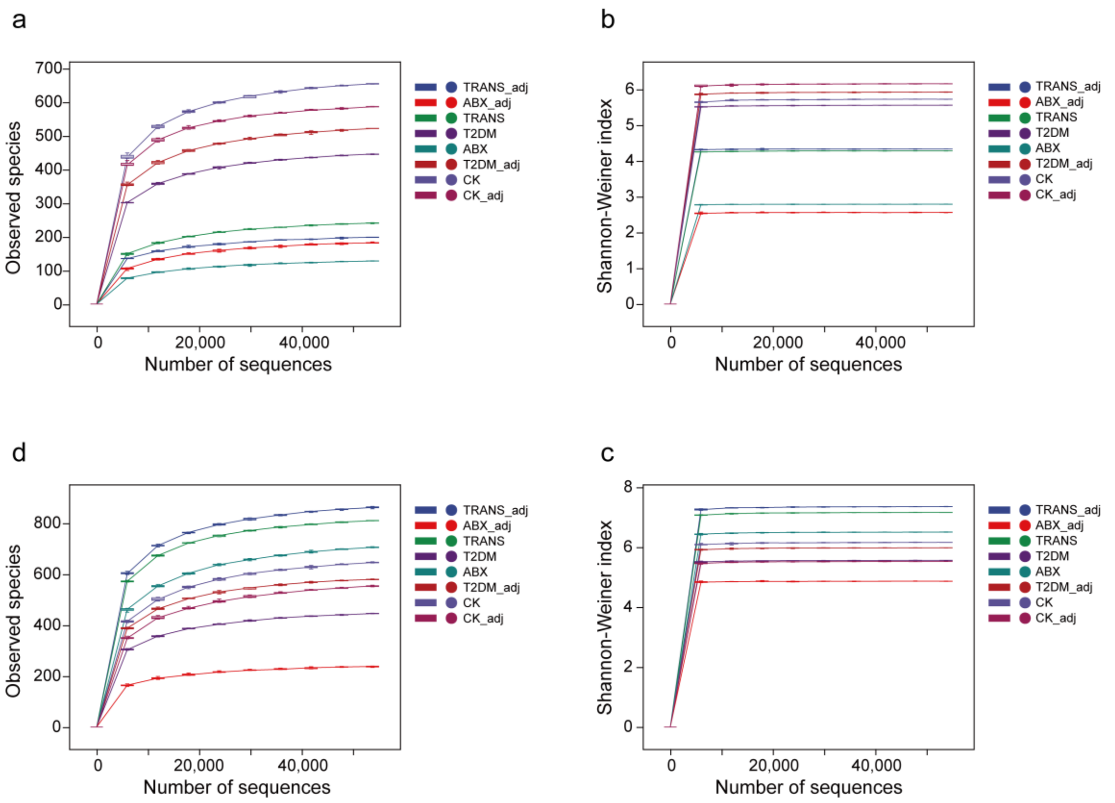

Figure S2. The rarefaction curves of fecal microbiota of day 0 (a and b) and gut microbiota of day 28 (c and d).

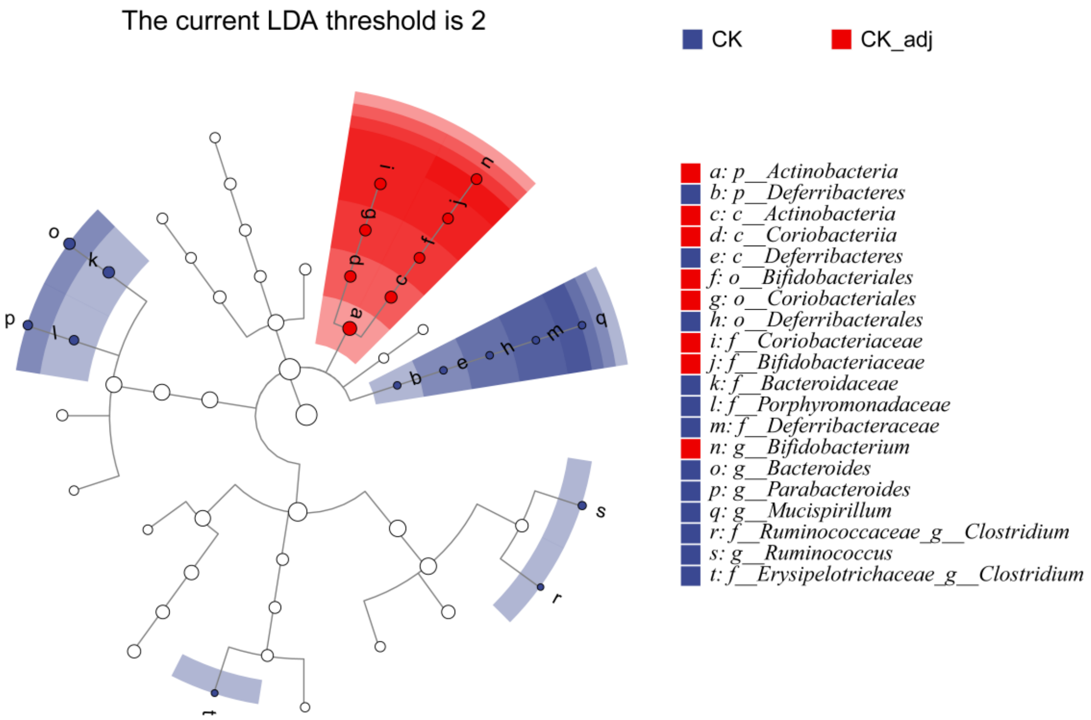

Figure S3. The cladogram of LEfSe analyses between CK and CK_adj groups.

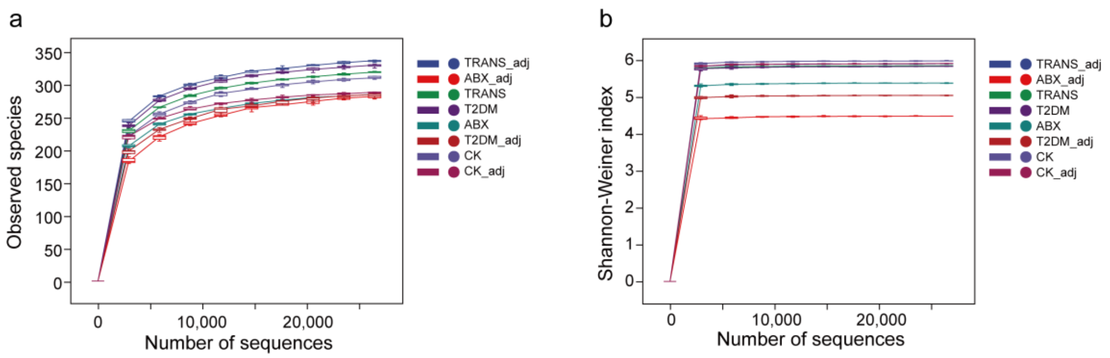

Figure S4. The rarefaction curves of lung microbiota.

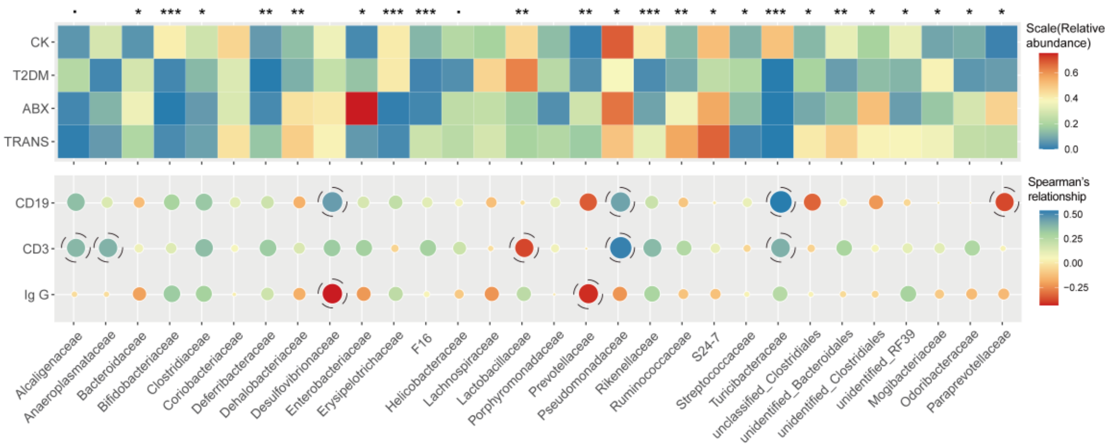

Figure S5. At family level (top 30), the relative abundance and relationships of gut microbiota (day28) with IgG level, CD3 and CD19. Kruskal–Wallis tests were employed to analyze the differences among four groups. ***: $P < 0.001$; **: $P < 0.01$; *: $P < 0.05$; ·: $P < 0.1$. Spearman's correlation analyses were employed to analyze the relationships of gut microbiota with IgG level, CD3 and CD19. Red color stand for negative relationship. Blue color stand for positive relationship. The bigger the circle is, the greater the correlation coefficient is. Black dashed circles represent for significant relationship ($P < 0.05$).

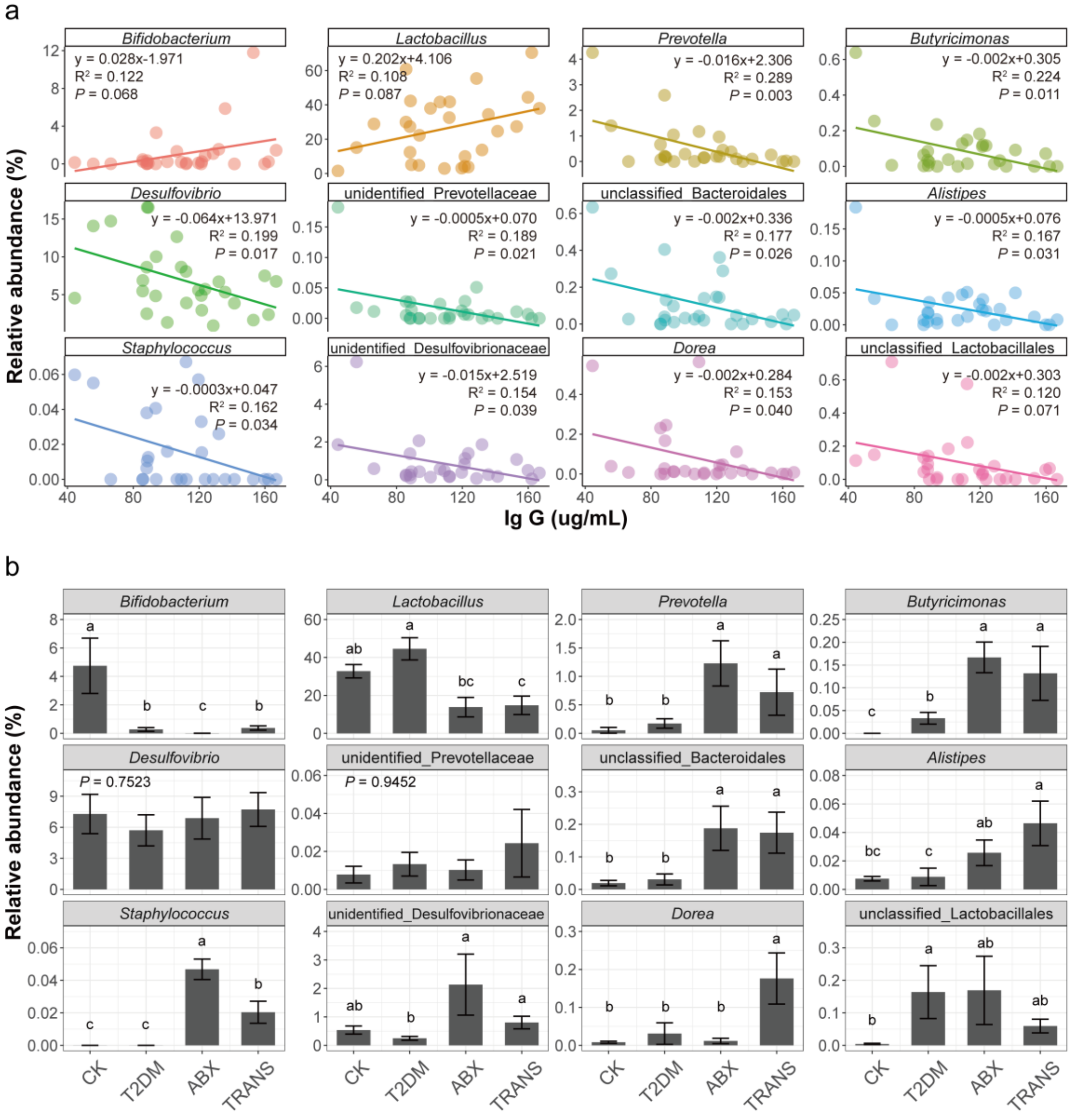

Figure S6. The genus that correlated with the level of Ig G of day 28 among the top 100 genus of gut microbes. a) The linear regression of the relative abundance of the genus and the level of Ig G of day 28. b) The comparison of the relative abundance of the genus (**Figure S6a**) among four groups (CK, T2DM, ABX, and TRANS) by using Kruskal–Wallis tests. Different letters indicate significant differences ($P < 0.05$).

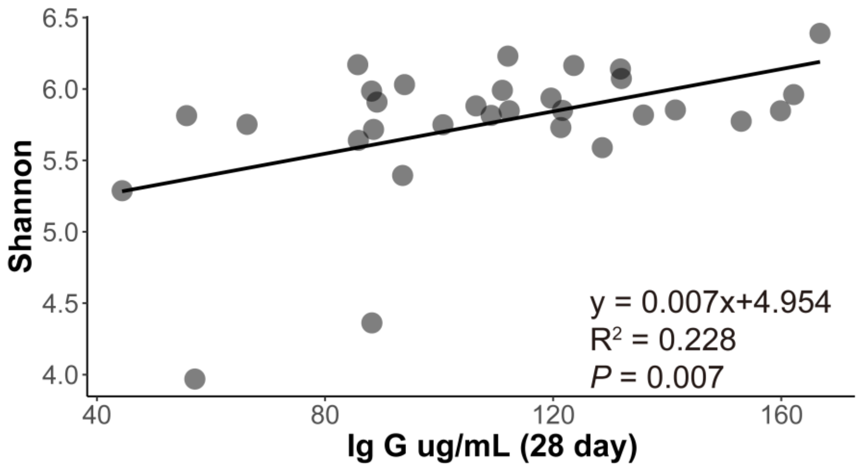

Figure S7. The linear regression of the Shannon index of lung microbiota and the IgG level of day 28.

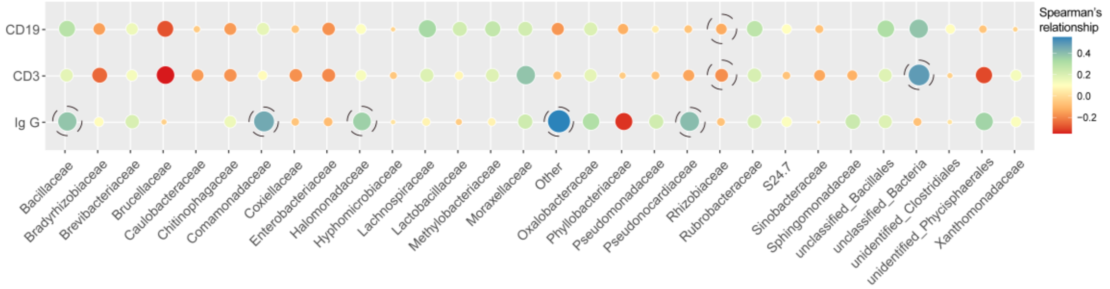

Figure S8. The Spearman's correlation between the relative abundance of lung microbiota (top 30 families) and the IgG level (day 28), CD19 and CD13. Red color stand for negative relationship. Blue color stand for positive relationship. The bigger the circle is, the greater the correlation coefficient is. Black dashed circles represent for significant relationship ($P <$ 0.05).

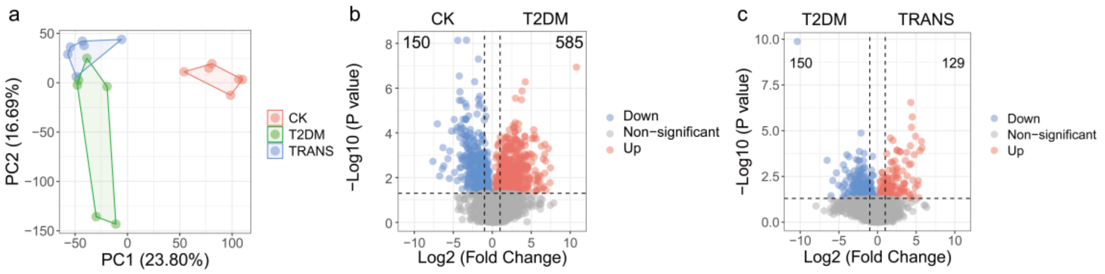

Figure S9. The PCA plot and the volcano plot (b: CK and T2DM groups; c: T2DM and TRANS groups) of gut metabolome.

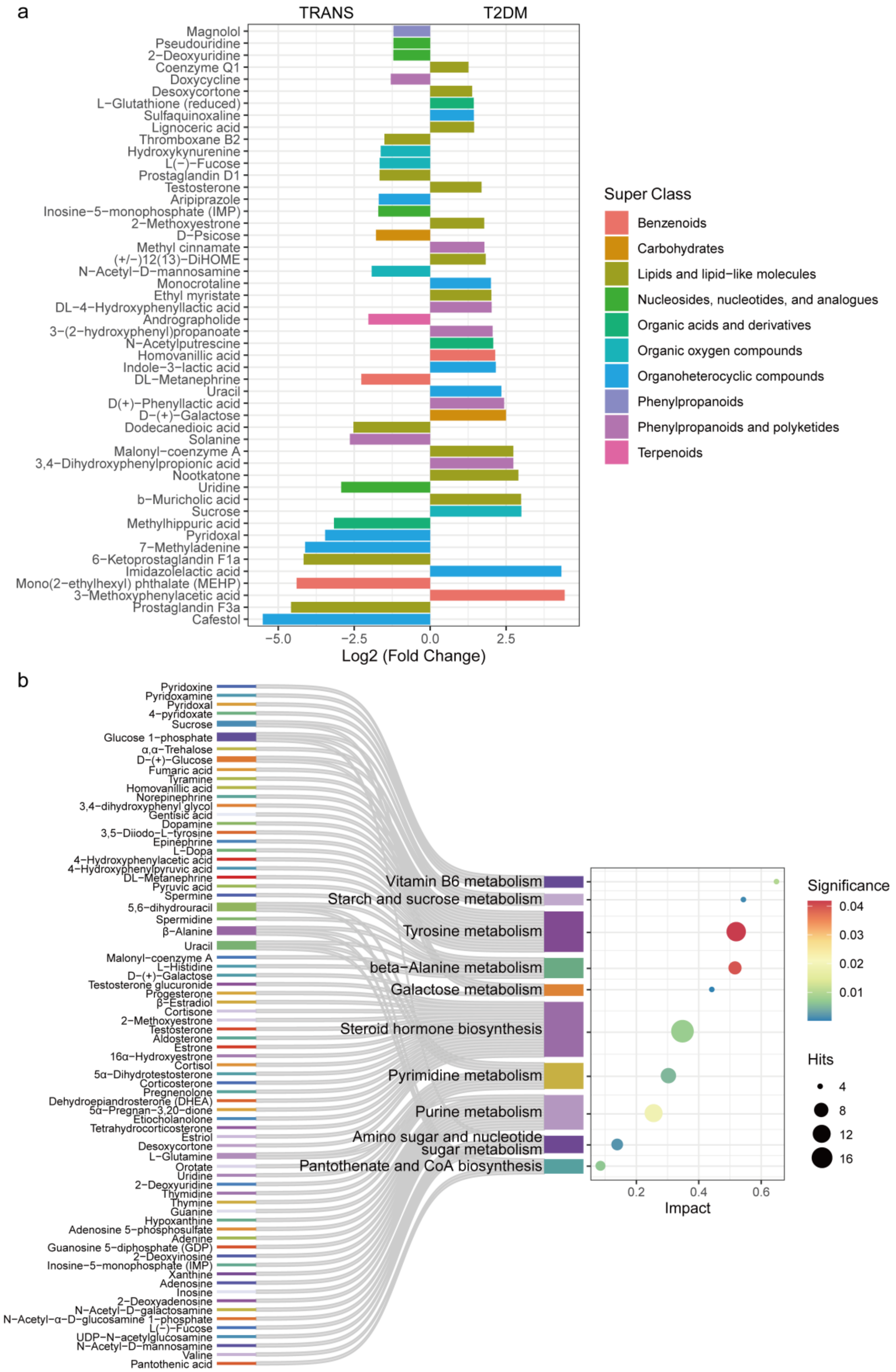

Figure S10. a) Significantly altered top 50 metabolites between TRANS and T2DM groups. b) Significantly changed top 10 pathways between TRANS and T2DM groups.

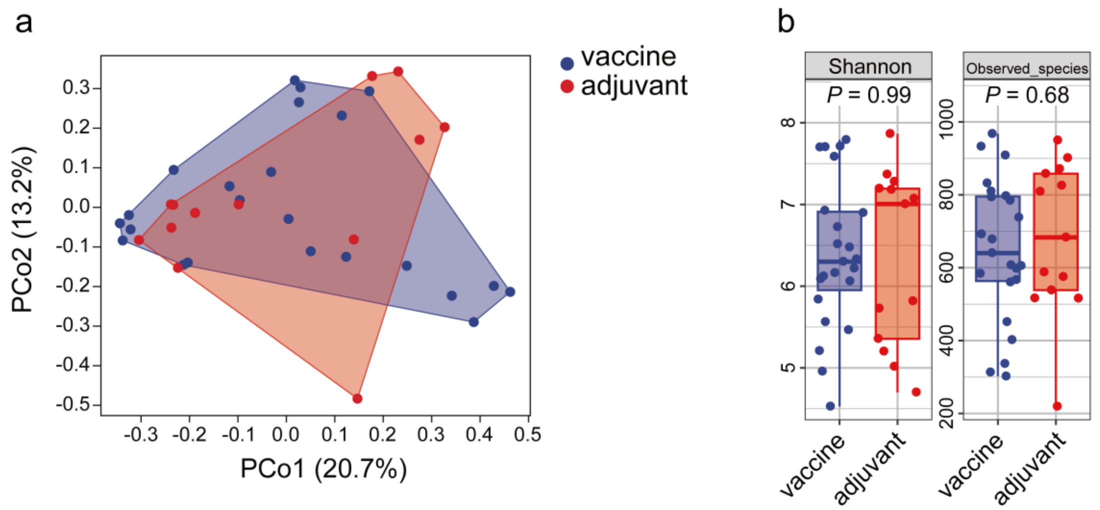

Figure S11. a) The PCoA of gut microbiota based on Bray-Curtis distance. b) The boxplot of Shannon index and observed species of gut microbiota. Wilcoxon rank sum tests were employed to analyze the differences between vaccine group and adjuvant group. Vaccine group represents for all the mice received SARS-CoV-2 vaccine. Adjuvant group represents for all the mice received adjuvant.

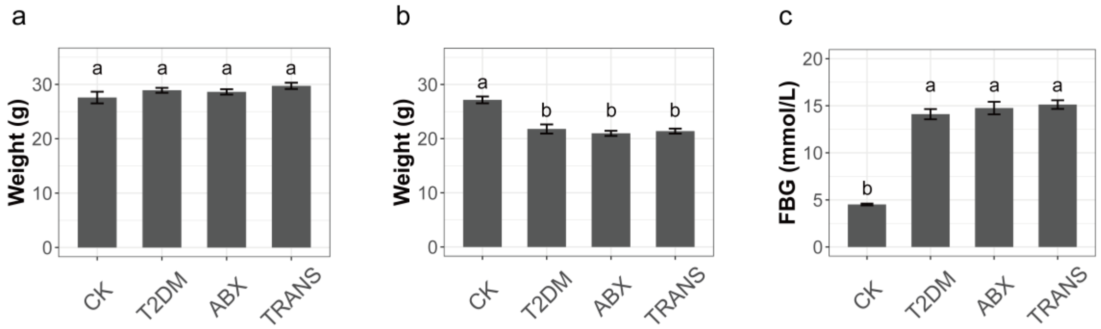

Figure S12. The body weight and fasting blood glucose (FBG) of four groups (CK, T2DM, ABX and TRANS). a) The body weight before modeling. b) The body weight after modeling. c) The FBG after modeling. Different alphabets stand for significant differences ($P < 0.05$) using Kruskal–Wallis tests.
