# Supplementary material for: Exploring the associations between gut microbiota composition and SARS-CoV-2 inactivated vaccine response in mice with type 2 diabetes mellitus
Source: mSphere. 2024 Aug 27;9(9):e00380-24. doi: 10.1128/msphere.00380-24 (PMC11423585; doi:10.1128/msphere.00380-24)
Supplement: Supplemental tables — Tables S1 to S7. [file msphere.00380-24-s0002.pdf]

Exploring the associations between gut microbiota composition and SARS-CoV-2 inactivated vaccine response in mice with type 2 diabetes mellitus

Long Liu[#1, 3, 4], Xianzhen He[#1, 2], Jiaqi Wang[1], Moran Li[1], Xiuli Wei[1], Jing Yang[1], Gong Cheng[5], Weixing Du*[1, 3], Zhixin Liu*[1, 3, 4], Xiao Xiao*[1, 3, 4]

[1] Department of Pathogen Biology, School of Basic Medical Sciences, Hubei University of Medicine, Shiyan, China; Department of Infectious Diseases, Renmin Hospital, Hubei University of Medicine, Shiyan, Hubei 442000, PR China

[2] Department of Children's Medical Center, Renmin Hospital, Hubei University of Medicine, Shiyan, China

[3] Institute of Virology, Shiyan Key Laboratory of Virology, Hubei University of Medicine, Shiyan, China

[4] Hubei Key Laboratory of Embryonic Stem Cell Research, Hubei University of Medicine, Shiyan, China

[5] New Cornerstone Science Laboratory, Tsinghua-Peking Joint Center for Life Sciences, School of Basic Medical Sciences, Tsinghua University, Beijing, China

# These authors contributed equally to this article.

* Corresponding authors.

Xiao Xiao, xiao910512@outlook.com

Zhixin Liu, lzx20022456@126.com

Weixing Du, duwx-025@163.com

Running title: Gut microbiota and SARS-CoV-2 vaccine response in mice

Table S1. The number of raw reads and clean reads of fecal microbiota and gut microbiota.

	Group	Raw reads		Clean reads	
		Total	Mean	Total	Mean
Fecal microbiota of day 0	CK	992649	99574	644062	64468
	T2DM	1639532	108281	1159388	74822
	ABX	1651176	119308	1472674	107099
	TRANS	1897247	118470	1365919	84810
	Total	6180604	111408	4642043	82799
Gut microbiota of day 28	CK_adjuvant	498670	124668	331913	82978
	CK	625795	125159	428201	85640
	T2DM_adjuvant	502794	125699	339022	84756
	T2DM	902146	112768	594419	74302
	ABX_adjuvant	202016	101008	134785	67393
	ABX	641071	128214	421980	84396
	TRANS_adjuvant	546368	109274	377637	75527
	TRANS	1107841	110784	744443	74444
	Total	5026701	117197	3372400	78680

Table S2. The number of raw reads and clean reads of lung microbiota.

Group	Raw reads		Clean reads	
	Total	Mean	Total	Mean
CK_adj	389931	97483	193361	48340
CK	535465	89244	331318	55220
T2DM_adj	374096	93524	246351	61588
T2DM	931333	116417	570986	71373
ABX_adj	298454	99485	173822	57941
ABX	559461	79923	352775	50396
TRANS_adj	536718	107344	320805	64161
TRANS	931333	93133	570986	57099
Total	4556791	97069	2760404	58265

Table S3. The comparison of alpha diversity of lung microbiota between CK and CK_adj groups by using Wilcoxon rank sum test.

	Mean of CK	Mean of CK_adj	*P* value
Observed species index	311.700	288.375	0.2381
Shannon-Weiner index	5.981	5.909	0.4571

Table S4. PERMANOVA analysis of lung microbiome between different groups (CK, CK_adj).

formula = distance matrix ~ group (CK, CK_adj), permutation =999

WUF	Df	Sum of Sqs	R^2	F	*P* value
group	1	0.002873	0.02414	0.1979	1
Residual	8	0.116119	0.97586		
Total	9	0.118992	1		
UUF	Df	Sum of Sqs	R^2	F	*P* value
group	1	0.12758	0.08554	0.7483	0.957
Residual	8	1.36385	0.91446		
Total	9	1.49143	1		

Table S5. The comparison of the relative abundance of lung microbes at phylum level between CK and CK_adj by using Wilcoxon rank sum test.

Taxon	Mean of CK	Mean of CK_adj	*P* value
Proteobacteria	0.694889	0.720318	0.619
Firmicutes	0.091074	0.077503	0.619
Actinobacteria	0.086493	0.083133	0.381
Bacteroidetes	0.032093	0.025597	0.08571
Planctomycetes	0.007845	0.00608	0.381
Other	0.006213	0.001934	0.8714
Armatimonadetes	0.003687	0.004559	0.619
Acidobacteria	0.003674	0.002985	0.2381
Cyanobacteria	0.003262	0.00325	0.3048
Chlamydiae	0.002235	0.003064	0.9429

Table S6. The genera that significantly correlated with the level of Ig G of day 14 among top 40 genus of fecal microbiota of day 0 by using Spearman's correlation analyses.

	r	*P*
unclassified_Enterobacteriaceae	-0.65	< 0.0001
unidentified_S24.7	0.53	0.0014
Desulfovibrio	0.50	0.0028
unidentified_Lachnospiraceae	0.45	0.0077
Oscillospira	0.54	0.0011
Acinetobacter	-0.47	0.0051
Adlercreutzia	0.49	0.0034
unidentified_Ruminococcaceae	0.56	0.0006
Ruminococcus	0.44	0.0095
Allobaculum	0.54	0.001
Oceanicaulis	-0.5	0.0028
Aliihoeflea	-0.69	< 0.0001
Nesterenkonia	-0.70	< 0.0001

Table S7. The genus that significantly correlated with the level of indole acrylic acid among top 50 genus of gut microbiota of day 28 by using Spearman's correlation analyses.

Genus	r	*P*
Ruminococcus	-0.7606	0.0002
Butyricimonas	-0.7219	0.0007
Vagococcus	-0.7008	0.0012
Lactobacillus	0.6966	0.0013
Allobaculum	0.6698	0.0024
Corynebacterium	-0.6486	0.0036
Prevotella	-0.6463	0.0038
Enterococcus	-0.6347	0.0047
Clostridium	0.5417	0.0202
